# Epigenetic Modifications and Therapy in Uveitis

**DOI:** 10.3389/fcell.2021.758240

**Published:** 2021-11-18

**Authors:** Yanli Zou, Jing Jing Li, Wei Xue, Xiangbin Kong, Hucheng Duan, Yiqun Li, Lai Wei

**Affiliations:** ^1^ Department of Ophthalmology, Affiliated Foshan Hospital, Southern Medical University, Foshan, China; ^2^ State Key Laboratory of Ophthalmology, Sun Yat-sen University, Guangzhou, China; ^3^ Department of Orthopaedics, Affiliated Foshan Hospital, Southern Medical University, Foshan, China

**Keywords:** uveitis, epigenetic regulation, DNA methylation, microRNAs, epigenetic therapy

## Abstract

Uveitis is a sight-threatening intraocular inflammation, and the exact pathogenesis of uveitis is not yet clear. Recent studies, including multiple genome-wide association studies (GWASs), have identified genetic variations associated with the onset and progression of different types of uveitis, such as Vogt–Koyanagi–Harada (VKH) disease and Behcet’s disease (BD). However, epigenetic regulation has been shown to play key roles in the immunoregulation of uveitis, and epigenetic therapies are promising treatments for intraocular inflammation. In this review, we summarize recent advances in identifying epigenetic programs that cooperate with the physiology of intraocular immune responses and the pathology of intraocular inflammation. These attempts to understand the epigenetic mechanisms of uveitis may provide hope for the future development of epigenetic therapies for these devastating intraocular inflammatory conditions.

## Introduction

Epigenetics refers to stable and heritable alterations in gene expression without involving changes in the nucleotide sequence and was first proposed by Dr. Waddington in the 1940s (Bird, 2007; Waddington, 2012). Epigenetic phenomena are of remarkable importance for gene expression patterns in normal physiological functions such as cellular development and differentiation, as well as in response to environmental factors (Jeffries, 2020). The epigenome serves as a critical interface between the environment and genome. It comes into play during development and works in a highly organized way to modulate the landscape of gene expression in various cell types (Guerrero-Preston et al., 2011). The epigenetic regulation of gene expression leads to typical growth through the dynamic transcription of gametogenesis in the embryonic and neonatal stages and lasts for a lifetime (Meissner et al., 2008; Guerrero-Preston et al., 2011). In the whole life cycle, in response to various endogenous and exogenous factors, epigenetic regulation undergoes various changes that are transient or permanent (Kaminsky et al., 2009; Grolleau-Julius et al., 2010; Romani et al., 2015). Disruptions in epigenetically controlled gene expression patterns can lead to autoimmune diseases, cancer and a variety of other diseases (Zhang et al., 2020a; Safi-Stibler and Gabory, 2020; Tzika et al., 2020). Over the past decade, the effects of epigenetic modifications on innate and adaptive immunity have been studied intensively, especially in autoimmune diseases (Brooks et al., 2010; Long et al., 2016; Agudelo Garcia and Berger, 2020).

Uveitis is serious intraocular inflammation that occurs worldwide and can lead to visual impairment and even blindness. Approximately 25% of the irreversible blindness is caused by uveitis and its complications in the developing countries (Nussenblatt, 1990; Suttorp-Schulten and Rothova, 1996; Rao, 2013). Uveitis frequently occurs among people aged 20–50 years and causes considerable economic burden (Rothova et al., 1992). The pathogenesis of uveitis has been considered to depend on complex interactions between multiple genetic substances and environmental risk factors. Genome-wide association studies (GWASs) have provided a powerful tool for the genome-wide analysis of genetic susceptibility to uveitis, revealing several genes associated with uveitis, including IL23R/C1ORF141, STAT4, and ADO/ZNF365/Egr2 (Hou et al., 2020). However, there are still some mechanisms that cannot be solved by genetics, and it is necessary to further study epigenetic modifications to explore the lack of heritability in uveitis risk (Hou et al., 2020).

In this Review, we introduce the main principles of epigenetic regulation, explain the possible association between epigenetic regulation and uveitis, and investigate how epigenetics could contribute to the understanding of the complex pathogenesis of uveitis. Finally, treatments for uveitis by manipulating epigenetic aberrations are discussed.

## Epigenetics and Uveitis?

Over the past 2 decades, twin studies have been performed to dissect the causal relationship between genetic and environmental factors in complex phenotypic traits and disease pathogenesis, including those in the eye (Montezuma et al., 2007; Sanfilippo et al., 2010; Tan et al., 2015). With the exception of a few cases, no consistency of uveitis has been reported between identical twins, implying that environmental factors associated epigenetic alterations may be important in the initiation of uveitis. Stress, lifestyle, nutrition/diet, and behaviors have been reported to change the susceptibility to disease induction (Sobrin and Seddon, 2014). At cellular level, there is considerable evidence that CD4^+^ T cell-dependent immune responses are important in the pathogenesis of uveitis (Lee et al., 2014). Of interest, the activation and differentiation of CD4^+^ T cells are typically epigenetic processes. In the process of differentiation from naive T cells into Th1, Th2, Th17, Treg and Tfh cells, the cell volume increases significantly, the morphology changes significantly, and the ability to secrete various cytokines and express immune functional proteins is obtained. It should be noted that during these processes there is no change in DNA sequence. On the other hand, epigenetic phenomena such as DNA methylation and histone modifications that occur during the regulation of chromatin recombination are key molecular bases for the major changes in gene expression and CD4^+^ T cell function (Wei et al., 2009; O’Shea and Paul, 2010). Therefore, we must seriously consider the role of epigenetics in the pathogenesis of uveitis.

## Environmental Risk Factors Underlying Epigenetic Modifications in Uveitis

BD and VKH syndrome have been proposed to be initiated by environmental factors in genetically predisposed individuals (Xu et al., 2021); however, the role of environment-cued epigenetic modifications in uveitis has not been studied carefully. The epigenetic machinery is widely influenced by the environment, as well as the environmental microbiome, which affects the host microbiome. In addition to seasonality, allergies and vitamin D being known environmental risk factors for uveitis in juvenile idiopathic arthritis (Clarke et al., 2021), emerging evidence has shown that host microbiomes, especially gut microbiomes, can influence the progression of uveitis (Horai and Caspi, 2019).

Both infectious uveitis caused by bacteria, viruses, fungi, or parasites and noninfectious uveitis are associated with a series of dysregulations in inflammatory genes and microRNAs (Wei et al., 2020). The expression of these genes sometimes requires chromatin remodeling, which can be modulated by both commensal and pathogenic bacteria. For example, mycobacteria have been demonstrated to be able to interfere with chromatin remodeling and inhibit IFN-γ-induced gene expression (Arbibe, 2008). IFN-γ is one of the major cytokines released by CD4^+^ Th1/Th17 cells to activate inflammatory cascades and cause local tissue damage in uveitis (Diedrichs-Mohring et al., 2018). During bacterial infection, the expression of several inflammatory genes can also be modulated at the epigenetic level (Zur Bruegge et al., 2017). The molecular mechanisms underlying microbiome-induced epigenetic alterations have not yet been understood; however, a growing number of studies suggest that microbial metabolites may have strong chromatin-modulating effects (Krautkramer et al., 2017). Butyrate, a major product of gut microbial fermentation, has been demonstrated to inhibit histone deacetylases (HDACs) (Chang et al., 2014). Other short-chain fatty acids (SCFAs), such as lactate, acetate, and propionate, could also exert effects on host chromatin, implying that the gut microbiota may be an important regulator of host epigenetic events (Chang et al., 2014).

In recent decades, there has been ample evidence documenting a role for the gut microbiota in uveitis (Molzer et al., 2020). A dogma in ophthalmic research is that the intraocular environment is always sterile under physiological conditions. Nevertheless, emerging evidence argues against intraocular sterility. As the first and foremost finding, our group identified the presence of an intraocular microbiota via quantitative PCR, transmission electron microscopy (negative staining), direct culture, and high-throughput sequencing technologies (Deng et al., 2021). A disruption of the intestinal barrier or blood-retina barrier could result in the leakage of bacteria or their metabolites into the circulatory system and the eye, leading to subsequent chromatin modifications and altered inflammatory gene expression (Smith et al., 2013).

## Mechanisms of Epigenetic Regulation

Epigenetic mechanisms have been widely reviewed for their ability to regulate gene transcription and genomic stability. Epigenetics is a key factor in maintaining normal cell growth, development and differentiation (Esteller, 2007; Goldberg et al., 2007; Jones and Baylin, 2007; Peixoto et al., 2020). The term “epigenetics” can be summarized as follows: the meiosis/mitosis of gene expression can be genetically changed, which is related to environmental factors, but the base sequence in DNA does not change (Bird, 2007). Since genome-wide analyses cannot provide enough answers to explain the complex biological processes in autoimmune diseases in some cases, epigenetic modifications retain additional regulatory factors in immune responses (Moosavi and Motevalizadeh Ardekani, 2016). Recognizing the complexity of the interaction between epigenetic regulation and changes in the immune system in autoimmune diseases is a prominent challenge for discovering new potential therapeutic strategies. The main mechanisms of epigenetic regulation are DNA methylation and histone modifications. In addition, RNAs, such as microRNAs (miRNAs), are now considered an additional layer of gene expression regulation (Vukic et al., 2019).

### DNA Methylation

DNA methylation is one of the earliest and most studied epigenetic regulatory mechanisms (Okano et al., 1999; Wildner and Diedrichs-Mohring, 2020). Studies have shown that DNA methylation largely occurs on CpG nucleotides, and approximately 70–80% of CpG islands are methylated in mammals (Jabbari and Bernardi, 2004; Law and Jacobsen, 2010). DNA methylation is an epigenetic mechanism mediated by DNA methyltransferase (DNMT), in which a methyl group is added to the fifth carbon atom of the cytosine ring, with S-adenosine methionine (SAM) as the methyl donor. There are five members of DNA methyltransferases (DNMTs), DNMT1, DNMT2, Dnmt3a, DNMT3b and DNMT3L, which play different roles either in maintaining DNA methylation (DNMT1, DNMT2) or acting as *de novo* DNA methylators (Dnmt3a, DNMT3b and DNMT3L) (Deplus et al., 2002; Dawson and Kouzarides, 2012; Jones, 2012). DNMTs can interact with each other and participate in the addition and removal of DNA by methyl groups. DNA methylation can be removed passively or actively (Reik and Walter, 2001; Seisenberger et al., 2013). DNA methylation affects a variety of biological processes, such as transcriptional inhibition, reversible promoter silencing and chromosomal instability (Bjornsson et al., 2004; Vaissiere et al., 2008). Studies have linked altered levels of DNA methylation to a variety of autoimmune diseases (Liu et al., 2011; Calabrese et al., 2012; Qiu et al., 2017a; Qiu et al., 2017b).

### Histone Modifications

Histones are basic proteins that bind to DNA in the nuclei of eukaryotes, and they are one of the highly conserved gene families in eukaryotes. There are five major histone families in most eukaryotes: H1/H5, H2A, H2B, H3, and H4. Two copies of histones H2A, H2B, H3, and H4 constitute an octamer core, which is the core part of the nucleosome, and the function of H1 is to combine with linear DNA to help form higher-order structures (Wong et al., 1998; Jenuwein and Allis, 2001). A growing body of evidence has confirmed that at least 12 specific modifications occur on the N-terminal amino acid residues of histones that affect the binding of nucleosomes to DNA and the three-dimensional structure of chromosomes and regulate gene expression. The histone modification model is as follows: acetylation (lysine), methylation (lysine and arginine), sumoylation (lysine), phosphorylation (serine and threonine), ADP ribosylation, ubiquitylation (lysine), citrullination, butyrylation, crotonylation, proline isomerization, formylation, serotonylation, propionylation, and dopaminylation (glutamine) (Tan et al., 2011; Nfonsam et al., 2020). These posttranslational modifications play an important role in regulating gene expression, DNA repair, chromatin dynamics and genome stability (Berger, 2007; Kouzarides, 2007). Among them, histone acetylation is an important type of histone modification, which means that the improvement in gene transcription activity and epigenetic markers is related to dynamic chromatin changes. Histone acetylation refers to HAT activating gene transcription through the acetylation of lysine residues in histones, while HDAC deacetylates histones and inhibits gene transcription. Histone acetylation is catalyzed by histone acetyltransferase (HAT) and HDACs (He et al., 2018). In addition, many amino acid residues in histones can be methylated, and different types of methylation, such as monomethylation (me 1), dimethylation (me 2) and trimethylation (me 3), show a variety of valence states. Changes in histone methylation patterns may promote or inhibit gene expression (Geng et al., 2021). Different histone modification types also influence each other to jointly regulate the expression of specific genes.

### MicroRNAs

MicroRNAs are a class of noncoding small RNAs with a length of 18–23 nucleotides located in the intragenic and intergenic regions of the genome. They are responsible for regulating the expression of approximately 60% of protein-coding genes in the human genome at the translation level. There is increasing experimental evidence that miRNA genes are distributed throughout the genome (Ambros, 2001; Ling et al., 2013; Vega-Tapia et al., 2021). It is well known that microRNAs can regulate various biological processes, such as proliferation, differentiation, apoptosis, the immune response and homeostasis (Chamorro Petronacci et al., 2019; Ashrafizadeh et al., 2020a; Ashrafizadeh et al., 2020b). These small dynamic RNA molecules regulate gene expression by binding to the 3′-untranslated region (3′-UTR) of the target mRNA, resulting in posttranscriptional inactivation of the target gene through mRNA degradation or the inhibition of translation (Catalanotto et al., 2016).

The role of histone modification in major rheumatic diseases such as rheumatoid arthritis has been considered (Li et al., 2009; Pedre et al., 2011; Singhal et al., 2015; Aslani et al., 2017), but there are few studies on histone modifications in uveitis. Therefore, we mainly discuss the role of DNA methylation and microRNAs in uveitis (Table 1).

**TABLE 1:** Epigenetic alterations in Uveitis.

Disease	Epigenetic change	Sample	Outcome	References
Experimental autoimmune uveitis	DNA methylation	Retinas and the RPE-Choroid Complexes	Tbx21 and Rorc showed dynamic methylation changes during EAU	Qiu et al. (2018)
	MicroRNA	PBMCs	36 miRNAs were upregulated and 31 miRNAs were downregulated	Guo et al. (2015)
		Retina	Upregulated: miRNA-223, 142-5p, 142-3p, 21, 146a, 146b, 1949, 1188-3p and 193	Watanabe et al. (2016)
			Downregulated: miRNA-181a, 183*, 124* and 331	
		Spleen, lymph nodes, and eye tissues	Downregulated: rno-miR-30b-5p	Sun et al. (2018)
		Eye tissues	Upregulated: miRNA-142-5p and miRNA-21 Downregulated: miRNA-182	Ishida et al. (2011)
		CD4^+^ T cells	Upregulated: miR-155	Escobar et al. (2013)
		Th17 cells	Upregulated: Mir-223-3p	Wei et al. (2019)
Behçet’s disease	DNA methylation	Monocytes and CD4^+^ T cells	383 CpG sites in monocytes and 125 CpG sites in CD4^+^ T cells differentially methylated	Hughes et al. (2014)
		PBMCs and Neutrophil	Unmethylated (uCuC) Alu allele frequency increased in inactive BD patients	Yuksel et al. (2016)
		Blood	4,332 differentially methylated CpG sites were associated with BD and the most significant locus was located in the 5′UTR of FKBP5	Yu et al. (2019)
		CD4^+^ T cells	Hypomethylation of GATA3, IL-4 and TGF-β promoter	Zhu et al. (2017a)
		PBMCs	Hypomethylation of TLR4 promoter	Kolahi et al. (2020)
		PBMCs	Hypermethylation of IL-10 promoter	Alipour et al. (2018)
		PBMCs	Hypomethylation of IL-6 promoter	Alipour et al. (2020)
		PBMCs	Hypomethylation of TNF-α promoter	Aziz et al. (2020)
	MicroRNA	PBMCs	Upregulated: miR-155	Kolahi et al. (2018)
		PBMCs	Upregulated: miR-326	Jadideslam et al. (2019)
			Downregulated: miR-21, miR-146b	
		PBMCs	Upregulated: miR-3591-3p	Woo et al. (2016)
			Downregulated: miR-638, miR-4488	
		PBMCs and dendritic cells	Downregulated: miR-155	Zhou et al. (2012)
		CD4^+^ T cells	Downregulated: miR-23b	Qi et al. (2014)
		PBMCs	Upregulated: miR-25, miR-106b, miR-326, miR-93	Ahmadi et al. (2019)
			Downregulated: miR-146a, miR-155	
Vogt–Koyanagi–Harada Disease	DNA methylation	CD4^+^ T cells	Hypermethylation of GATA3, IL-4 and TGF-β promoter	Zhu et al. (2017b)
		monocyte-derived dendritic cells	Hypermethylation of IRF8	Qiu et al. (2017a)
	MicroRNA	Serum	153 upregulated and 35 downregulated	Asakage et al. (2020)
		CD4^+^ T cells	Downregulated: miR-20a-5p	Chang et al. (2018)
Anterior uveitis	MicroRNA	PBMCs	Downregulated: miRNA146a, miRNA155 and miRNA125a5p	O'Rourke et al. (2019)
Experimental autoimmune anterior uveitis	MicroRNA	Iris, ciliary bodies, and popliteal lymph nodes	Upregulated: miR-182-5p, miR-183-5p and miR-9-3p	Hsu et al. (2015)
			Downregulated: miR-146a-5p, miR-155-5p, miR-147b and miR-223-3p	

## Epigenetic Modifications in Different Types of Uveitis

### Epigenetic Modifications and Experimental Autoimmune Uveitis

Experimental autoimmune uveitis (EAU) is an animal model of human uveitis that has been extensively used in preclinical research (Caspi et al., 1988; Nussenblatt, 1991). Interphotoreceptor retinoid-binding protein (IRBP), a specific retinal antigen, can be used to induce EAU in mice (Caspi et al., 2008). In a highly susceptible B10R.III mouse strain, IRBP 161–180 peptide-induced EAU showed severe inflammation, including inflammation in the anterior and posterior segments of the eye, very similar to the clinical abnormalities observed in human panuveitis (Jiang et al., 1999).

T helper cell subsets and their featured transcription factors are related to various autoimmune diseases (Rahimi et al., 2019; Sakaguchi et al., 2020). To investigate whether the methylation of these main transcription factors is related to the development of EAU, Qiu Y et al. investigated whether the methylation of the T cell transcription factors TBX21 and RORC changed significantly during the development of EAU. They also provided evidence that DNMT1 may play an important role in regulating the DNA methylation of these two transcription factors (Qiu et al., 2018).

Mounting evidence has also demonstrated that certain miRNA imbalances are related to the progression of EAU. In EAU rats, it was shown that 36 miRNAs were upregulated, and 31 miRNAs were downregulated, all of which are closely associated with immune signaling pathways (Guo et al., 2015). Watanabe et al. found that the expression of 9 miRNAs (miR-223, 142-5p, 142-3p, 21, 146a, 146B, 1949, 1188-3p and 193) was significantly increased and that the expression of 4 miRNAs (miR-181a, 183 *, 124 * and 331) was decreased in the retinas of EAU rats on the 14th day after immunization. Among them, the expression of miR-223 and miR-146a was consistent with the increase in IL-1β/McP-1 in the eye with EAU (Watanabe et al., 2016). Furthermore, Sun et al. demonstrated that rno-miR-30b-5P expression was decreased in the spleen, lymph nodes and eye tissues of EAU rats, and rno-miR-30b-5P played a role in the pathogenesis of uveitis by directly regulating the levels of IL-10- and TLR4-positive cells to affect the development of uveitis (Sun et al., 2018). Th17 cells play a key role in the pathogenesis of autoimmune uveitis (Peng et al., 2007). Several studies have revealed that miRNAs influence the pathogenesis of EAU by regulating the Th17 cell response. Significantly increased levels of miR-142-5p and miR-21 were detected in mouse eye tissues 7 days after EAU induction, while significantly decreased levels of miR-182 were detected. The dynamic changes in these miRNAs were similar to those of IL-17. These results suggest that miRNAs regulate EAU development by influencing IL-17 expression (Ishida et al., 2011). In addition, Escobar et al. demonstrated that STAT3 directly binds to the miR-155 locus and forms a STAT3 and miR-155 axis that amplifies pathogenic Th17 cells and exacerbates EAU inflammation (Escobar et al., 2013). In another study, miR-223-3p was confirmed to be significantly upregulated in interphotoreceptor retinoid-binding protein-specific Th17 cells. Mechanistic studies showed that miR-223-3p directly inhibited the expression of FOXO3, which negatively regulated the pathogenic Th17 response partially by inhibiting il-23R expression (Wei et al., 2019). Together, these results suggest that miRNAs play an important role in the pathogenesis of EAU.

### Epigenetic Modifications and Behcet’s Disease

BD is a chronic multisystem disease characterized by recurrent inflammation, and its underlying histopathology is occlusive vasculitis (Mendes et al., 2009). It is characterized by ocular lesions, oral ulcer, genital ulcer, and multiple skin lesions (Mishima et al., 1979; Yang et al., 2008; Zeidan et al., 2016). The ocular involvement is found in 83–95% in males and 67–73% in females, and BD is the cause of blindness in about 12% of acquired blindness in adults. (Mishima et al., 1979). The eye involvement mainly manifests with chronic, recurrent bilateral non-granulomatous uveitis with necrotizing retinal vasculitis (Davatchi et al., 2017), which may lead to severe vision loss (Park et al., 2014; Ksiaa et al., 2019). According to the most widely accepted criteria for BD published by the international research group (ISG) in 1990, uveitis is an important diagnostic criterion for (Criteria for diagnosis of Behcet’s disease, 1990). Furthermore, BD and VKH disease are the most common non-infectious uveitis entities seen in Asia (Pineton de Chambrun et al., 2012; Hou et al., 2020). Although there is increasing evidence about the pathogenesis of BD, its actual etiology is still unclear. Previous studies have shown that genetic and epigenetic factors play an important role in BD. The incidence and clinical manifestations of BD are related to epigenetic factors such as age, sex and smoking, as well as exogenous factors such as diet, infection and stress (Saadoun and Wechsler, 2012; Demirelli et al., 2015; Alipour et al., 2018; Farhadi et al., 2019).

The first epigenome-wide study in BD was carried out by Hughes T et al. In their study, they analyzed the genome-wide DNA methylation patterns of monocytes and CD4^+^ T cells and found significantly different methylation sites between untreated BD patients and controls: 383 CpG sites in 228 genes in monocytes (129 hypermethylated and 254 hypomethylated) and 125 CpG sites in 62 genes in CD4^+^ T cells (67 hypomethylated and 58 hypermethylated). Furthermore, they performed bioinformatics analysis to reveal the abnormal DNA methylation pattern between genes that regulate cytoskeleton dynamics and indicated that the multiclass structure of the cytoskeleton and the abnormal DNA methylation of regulatory proteins may contribute to the pathogenesis of BD (Hughes et al., 2014). In another study, Yüksel et al. evaluated the epigenetic changes of interspersed repetitive sequences (IRSs) in BD using combined bisulfite restriction analysis-interspersed repetitive sequences (COBRA-IRS). They found that the unmethylated (uCuC) Alu allele frequency increased in the peripheral blood mononuclear cells (PBMCs) and neutrophils of inactive BD patients, while the hypomethylation frequency did not differ significantly between active BD patients and controls. Therefore, the pathogenesis of BD may involve changes in IRS element methylation levels (Yuksel et al., 2016). Yu H et al. found 4,332 differentially methylated CpG sites associated with BD in a recent genome-wide DNA methylation profile study. Further validation experiments showed that the most significant locus was located in the 5′-UTR of FKBP5 (cg03546163, *p* = 3.81E-13) (Yu et al., 2019). In addition, Zhu Y et al. revealed that the hypermethylation of GATA3 and TGF-β may lead to gene transcriptional silencing, which may play a role in the pathogenesis of BD (Zhu et al., 2017a). Kolahi S et al. suggested that hypomethylation of the TLR4 gene may be involved in the pathogenesis of BD by increasing TLR4 expression (Kolahi et al., 2020). Alipour S et al. found that the expression level of the IL-10 gene in BD patients was significantly decreased, while the promoter methylation rate in patients with low IL-10 mRNA expression was significantly higher than that in controls (Alipour et al., 2018). In contrast, the expression of the IL-6 and TNF-α genes was significantly increased in BD patients, and the methylation levels of the IL-6 promoter and TNF-α were significantly decreased (Alipour et al., 2020; Aziz et al., 2020). Combing these data suggests that DNA methylation is involved in the development of BD. Of note, all of the BD patients in the studies carried out by Yuksel et al. (2016), Yu et al. (2019), Zhu et al. (2017a) had uveitis. In the other studies, such as studies performed by Hughes et al. (2014), Kolahi et al. (2020), Alipour et al. (2018), Alipour et al. (2020), Aziz et al. (2020), only part of the BD patients had ocular symptoms which presumably involved uveitis.

MicroRNA expression disorders have been widely studied in BD patients, and some miRNAs are considered biomarkers for disease diagnosis. For example, Kolahi et al. found in the PBMCs of BD patients with uveitis in Iran that the expression of miR-155 was significantly increased compared with that in healthy volunteers, and there was no significant difference in the expression of miR-146a (Kolahi et al., 2018). In addition, Jadideslam et al. confirmed that the expression of miR-21 and miR-146b decreased significantly in BD patients from Iran, while the expression of miR-326 increased significantly. They proposed that the expression rate of miR-326 could be used as a biomarker to predict uveitis and severe ocular involvement in BD patients (Jadideslam et al., 2019). Moreover, Woo et al. observed that the expression of miR-638, miR-4488, and miR-3591-3p in the PBMCs of BD patients, part of them suffered from ocular symptoms, was altered, which is associated with the production of IL-6, a pleiotropic cytokine implicated in the pathogenesis of many immune-mediated disorders including several types of non-infectious uveitis (Woo et al., 2016). Uveitis associated with BD and VKH syndrome is likely also involved (Perez et al., 2004; Mesquida et al., 2014; Lin, 2015).

The inflammatory response, especially the Th17 cell-mediated inflammatory response, is the basis of BD (Chi et al., 2008; Hamzaoui, 2011; Alpsoy, 2016; Leccese and Alpsoy, 2019). Studies have shown that miRNAs are key modulators of the immune response in BD, and the regulation of Th17 cell activity has been the subject of miRNA studies in BD. miR-155 was found to inhibit dendritic cell-driven Th17 responses by targeting TAB2 in BD (Zhou et al., 2012). In addition, the balance of Th1/Th17/Treg cells and their transcription factor-related microRNAs has attracted attention in BD patients. For example, miR-23b levels in the CD4^+^ T cells of patients with active BD decreased significantly, accompanied by increased Notch pathway activation and an active Th1/Th17 response (Qi et al., 2014). In another report, Ahmadi et al. observed that the proportion of Treg cells decreased significantly and the proportion of Th17 cells increased significantly in BD patients, along with significant upregulation of miR-25, miR-106b, miR-326 and miR-93 and downregulation of miR-146a and miR-155 (Ahmadi et al., 2019). In addition, all of the BD patients suffered from uveitis in the studies carried out by Zhou et al. (2012), Qi et al. (2014) and part of patients had eye involvement in the study performed by Ahmadi et al. (2019).

Given that the coding sequences of microRNAs are affected by genetic variation, similar to any other gene (Cammaerts et al., 2015), the genetic variation in BD has also been studied. Several such variants have been identified in patients with BD, such as rs2910164 (miR-146a), rs11614913 (miR-196a2), rs3746444 (miR-499), and rs76481776 (miR-182) (Qi et al., 2013; Yu et al., 2014; Zhou et al., 2014; Oner et al., 2015; Ibrahim et al., 2019; Kamal et al., 2021). Zhou et al. were the first to identify a strong association between rs2910164 of miR-146a and BD in the Chinese population, and the expression of miR-146a, interleukin (IL-17), tumor necrosis factor (TNF)α and IL-1β was decreased in individuals carrying the CC genotype (Zhou et al., 2014). Oner et al. proved that the homozygous CC genotype and C allele of the rs2910164 polymorphism are protective factors against BD in Turkey. In addition, they found that the miR-499 rs3746444 homozygous (TT) genotype significantly increased the risk of BD in individuals in Turkey (Oner et al., 2015). Additionally, Kamal et al. found that the miR-146a rs2910164 variant plays an important role in the development and clinical regulation of BD in Egyptian patients (Kamal et al., 2021). Moreover, the mutation of miR-196A2/RS11614913 is associated with the risk of BD by reducing the expression of miR-196a and increasing the secretion of the target protein Bach1 as well as the proinflammatory IL-1β and McP-1 cytokines (Qi et al., 2013). In addition, Yu et al. revealed that the frequency of the miR-182/rs76481776 CC genotype and C allele was significantly reduced in BD patients (Yu et al., 2014). MiRNAs and their single nucleotide polymorphisms affect BD-related cells and molecules, deepen our understanding of the pathogenesis of BD, and provide new ideas for the diagnosis and treatment of BD. Furthermore, all of the BD patients had uveitis in the studies carried out by Zhou et al. (2014), Qi et al. (2013), Yu et al. (2014) and part of BD patients had eye involvement in the studies performed by (Oner et al. (2015), Kamal et al. (2021).

### Epigenetic Modifications and Vogt–Koyanagi–Harada Disease

VKH syndrome is an autoimmune disease with multiple system involvement. The clinical changes are characterized by bilateral granulomatous panuveitis accompanied by other system damage, such as vitiligo, hearing loss and nervous system damage (Yang et al., 2007; Yang et al., 2019). However, in the early stage of the disease, patients usually show isolated ocular involvement, the choroid is the main site of ocular inflammation, and the iris and ciliary body may also be involved (Baltmr et al., 2016). At this stage, patients mainly complain of visual impairment, and most patients with bilateral posterior uveitis often manifest with severe retinal detachment, congestion and edema of the optic nerve papilla, posterior choroidal thickening, and an increased retinal choroid layer around the optic nerve papilla (Du et al., 2016). The exact molecular mechanism of VKH disease is still not known and needs further study.

Zhu Y et al. found that the promoter methylation levels of GATA3, IL-4 and TGF-β were remarkably increased in patients with active VKH (Zhu et al., 2017b). In another study, they found that decreased IRF8 mRNA expression in monocyte-derived dendritic cells (DCs) of patients with active VKH was associated with higher methylation levels (Qiu et al., 2017a).

In a recent study, Asakage et al. conducted advanced high-throughput, untargeted and unbiased comprehensive miRNA analysis using serum samples from patients with noninfectious uveitis. The results showed 153 upregulated miRNAs and 35 downregulated miRNAs in VKH patients, and let-7g-3p was determined to be the best predictor miRNA of VKH (Asakage et al., 2020). Other microRNAs have also been reported to be associated with VKH disease. miR-20a-5p was found to be expressed at low levels in CD4^+^ T cells in patients with active VKH (Chang et al., 2018). Copy number variations in miR-146a, miR-23a, and miR-301a have been revealed to confer risk for VKH syndrome (Hou et al., 2016). Furthermore, miR-182 has also been confirmed to be involved in the genetic susceptibility of VKH (Yu et al., 2014). VKH is a complex disease whose pathogenesis is not fully understood. Although scant, evidence supports a role for miRNAs in the development of VKH disease.

### Epigenetic Modifications and Anterior Uveitis

Anterior uveitis (AU) is the most common form of uveitis according to the majority of surveys worldwide and presents in up to 90% of cases of uveitis (Chang and Wakefield, 2002; Wakefield and Chang, 2005). AU is the most common presentation among patients with ankylosing spondylitis (AS), occurring in 90% of all uveitis cases in spondylitis (SpA) (Rosenbaum and Chandran, 2012). It is frequently characterized by sudden onset and is often unilateral or unilateral alternating, anterior and recurrent (Zeboulon et al., 2008; Canouï-Poitrine et al., 2012). Anterior uveitis associated with SpA is typically a nongranulomatous type of uveitis characterized by the presence of fine keratic precipitates visible on slit lamp examination of the anterior segment. Intraocular pressure is usually low due to severe inflammation of the ciliary body. In severe forms of acute AU (AAU), hypopyon and fibrin can be visualized as a white and dense clot in the anterior chamber (Agrawal et al., 2010). In addition, approximately 50% of all patients with acute AU (AAU) are human leukocyte antigen B27 (HLA B27) positive (Wakefield et al., 2020). Some authors speculate on the prognosis of HLA-B27-related AAU, reporting a higher frequency of recurrence and a worse outcome than HLA-B27-negative AAU (Rothova et al., 1987; Power et al., 1998). However, in a study based on meta-analysis conducted by D ′Ambrosia and colleagues, they concluded that there is no significant difference between HLA B27 positive and HLA B27 negative AAU with regard to the final visual acuity and structural complications, such as posterior synechiae, cataract, and macular oedema (D'Ambrosio et al., 2017).

Although the exact pathogenesis of AU remains unclear, large-scale genome wide association studies have confirmed that AU is a polygenic disease, with overlaps with the seronegative arthropathies and inflammatory bowel diseases, associations that have been repeatedly confirmed in clinical studies (Wakefield et al., 2020). Some evidence has proven that epigenetic modifications are involved in the regulation of AU development, such as microRNAs. O'Rourke et al. demonstrated increased expression of miR-146a, miR-155 and miR-125a5p in the PBMC of AU patients compared with healthy controls. The expression of miR-155 was increased following TLR1/2 and TLR4 stimulation and the expression of miR-146a was increased in response to IL1β. In a proinflammatory environment, miR-155 overexpression in THP1 cells yielded increased cytokine output whereas miR-146a overexpression showed decreased cytokine output. CD80, PRKCE and VASN were confirmed as novel targets for miR-146a and SMAD2, TYRP1 and FBXO22 for miR-155 (O'Rourke et al., 2019). In another study, Verhagen et al. designed a strategy to robustly identify changes in the miRNA profiles of two independent cohorts totaling 54 untreated patients (with one of three archetypical types of noninfectious uveitis: HLA-B27–associated acute AU, idiopathic intermediate uveitis, or Birdshot uveitis and eye-restricted disease) and 26 age-matched controls. Using stringent selection criteria, they identified and independently validated a miRNA cluster that is associated with noninfectious uveitis. Pathway enrichment analysis for genes targeted by this cluster revealed significant enrichment for the PI3K/Akt, MAPK, FOXO, and VEGF signaling pathways, and photoreceptor development. In addition, unsupervised multidomain analyses linked the presence of the uveitis-associated miRNA cluster to a different composition of leukocyte subsets, demonstrating systemic changes in epigenetic regulation underlying noninfectious uveitis (Verhagen et al., 2018). Furthermore, low gene copy numbers of miR-143, miR-146a, miR-9-3, miR-205 and high gene copy numbers of miR-301a and miR-23a were associated with susceptibility to AAU + AS + patients. A low copy number of miR-146a and a high copy number of miR-23a and miR-205 were associated with AAU + AS- patients (Yang et al., 2017). Based on these observations, it was speculated that these differentially expressed miRNAs might contribute to the pathogenesis of AU, but the potential roles of miRNAs in the AU are still in the early discovery stage and need to be fully explored in the future.

Experimental autoimmune anterior uveitis (EAAU) is an animal model of idiopathic human anterior uveitis in which inflammation is limited to the anterior segment and does not affect retinal tissue and photoreceptor cells (Broekhuyse et al., 1991). The pathogenesis of EAAU is largely unknown, but epigenetic mechanisms provide evidence for a better understanding of the disease. Hsu et al. Observed the dynamic changes of miRNA during EAAU, the down regulation of mir-146a-5p, mir-155-5p, mir-223-3p and mir-147b, and the up regulation of mir-182-5p, mir-183-5p and mir-9-3p. Meanwhile, cytokine analysis showed that IFN- γ, IL-17, IL-12A, IL- 1 β and IL-6 were overexpressed and IL-10 was down regulated. Summarizing these results, they speculated that these differentially expressed miRNAs may promote the dynamic changes of Th1/Th17 related cytokines, so as to promote the pathogenesis of EAAU (Hsu et al., 2015). However, the research of miRNA in EAAU is still in infancy, and future exploration needs to be fully developed.

## Prospects for Epigenetic Therapy in Uveitis

In recent decades, research on the role of epigenetic regulation in health and disease has increased explosively. Specific epigenetic clinical markers have attracted the attention of scientists, and epigenetic therapy has been proposed. Many epigenetic drugs, such as DNA methylation inhibitors and HDAC inhibitors, have been approved by the U.S. Food and Drug Administration and have been used in clinical treatment or are currently being tested in clinical trials, especially those on cancer treatment and the prevention and treatment of autoimmune diseases (Walton, 2016; Prachayasittikul et al., 2017; Jones et al., 2019; Morel et al., 2020). With regard to autoimmune uveitis, epigenetic therapy has been studied in animal models and *in vitro* trials and is expected to be used in clinical trials in the future (Table 2). The animal model of uveitis, EAU, is helpful for studying the pathogenesis of this disease as well as to design and examine treatment strategies.

**TABLE 2:** Epigenetic therapies in experiments and animal model of Uveitis.

Drug	Mechanism of action	Consequence	References
Zebularine	DNMT inhibitor	Downregulation of IL-17 and IFN-γ expression repression the infiltration rate of inflammatory cells in intraocular of EAU	Zou et al. (2019)
Vorinostat	HDAC inhibitor	Repression the Th1 and Th17 cells as increase the Th0 and Treg cells. inhibition production of transcription factors, including STAT1, STAT3 and p65 in EAU.	Fang et al. (2016)
Curcumin	HDAC inhibitor	Suppression of inflammatory cytokines in M1 macrophages of BD patients	Palizgir et al. (2018)
miRNA mimic	miRNA replenishment	Targeting miR-155 leads to reduce TNF-α, IL-6, and IL-1β by affecting the Akt/mTOR signaling pathway and autophagy on DC form BD patients	Liang et al. (2021)
miRNA mimic	miRNA replenishment	Targeting miR-146a leads to reduce intraocular inflammation and leukocyte infiltration through the inhibition of NF-κB in EAAU.	Hsu et al. (2017)
anti-miRNA adenovirus	miRNA depletion	Targeting miR-21-5p leads to affect the balance of Th17 and Treg cells and reduce retinal cell apoptosis in EAU.	Shi et al. (2019)
miRNA mimic	miRNA replenishment	Targeting miR-182-5p leads to inhibit the pathogenic Th17 response by negatively regulation of TAF15 in EAU.	Zhang et al. (2020b)
miRNA mimic	miRNA replenishment	Targeting rno-miR-30b-5p leads to suppress IL-10 and TLR4 positive cell proportion and ameliorate the development of EAU.	Sun et al. (2018)

DNMT DNA, methyltransferase; HDAC, histone deacetylase.

In our previous study, we evaluated the efficacy of zebularine, a DNA methylation inhibitor with low cellular toxicity and a long half-life (Billam et al., 2010), in the course of EAU. Our results showed that zebularine inhibited the expression of the inflammatory cytokines IFN-γ and IL-17 in human and mouse CD4^+^ T cells *in vitro*. Importantly, zebularine also significantly reduced intraocular inflammation and retinal tissue damage in a mouse EAU model *in vivo*, indicating that zebularine is a new candidate therapeutic agent for uveitis (Zou et al., 2019).

In addition, studies have evaluated the potential therapeutic effects of HDAC inhibitors in EAU. In one study, Fang et al. observed that vorinostat alleviated the clinical and histopathological manifestations of EAU by inhibiting the production of Th1 and Th17 cells and increasing the production of Th0 and Treg cells. In addition, vorinostat treatment significantly reduced IFN-γ and IL-17A expression levels but increased IL-10 levels. Mechanistic studies showed that vorinostat therapy greatly inhibited transcription factors, including STAT1, STAT3 and p65. These data suggest that vorinostat may be a potential anti-inflammatory drug for the treatment of uveitis (Fang et al., 2016). In another study, curcumin, a natural phenolic compound with an inhibitory effect on acetyltransferase activity (Radomska-Lesniewska et al., 2019), was used to study macrophages in patients with BD. The results showed that curcumin could inhibit the expression and production of inflammatory cytokines in the M1 macrophages of BD patients, suggesting that curcumin could better regulate inflammatory signals in macrophages from BD patients compared with macrophages from HCs (Palizgir et al., 2018).

In view of the important roles of microRNAs in disease, miRNA-based therapies have been widely used, and miRNA mimics/inhibitors, lentiviral overexpression plasmids and other approaches have also been attempted for the treatment of uveitis (Li and Rana, 2014; Lu et al., 2019; Takahashi et al., 2019). In a recent study, Liang et al. found that the transfection of miR-155 mimics into dendritic cells (DCs) from patients with BD reduced the expression of TNF-α, IL-6, and IL-1β in DCs (Liang et al., 2021). In addition, Hsu et al. found that the intravitreal injection of an appropriate concentration of nucleic acid miR-146a simulant effectively inhibited the intraocular inflammation of experimental autoimmune anterior uveitis (Hsu et al., 2017). Shi et al. showed that the subretinal injection of anti-miR-21-5p adenovirus attenuated EAU by inhibiting the inflammatory response and reducing retinal cell apoptosis. Their results demonstrate that miR-21-5p can be used as a therapeutic target for uveitis and other autoimmune diseases (Shi et al., 2019). Furthermore, Zhang et al. demonstrated that miR-182-5p mimicry inhibited the pathogenic Th17 response in EAU mice by directly inhibiting the transcription promoter TATA-binding protein-related factor 15 (Zhang et al., 2020b). In addition, Sun et al. revealed that rno-miR-30b-5p mimics play a role in the pathogenesis of uveitis by reducing the number of IL-10- and TLR4-positive cells and influencing the development of uveitis (Sun et al., 2018). However, research on miRNAs for the treatment of autoimmune uveitis is in its early stages and requires broader investigations.

## Conclusion

Uveitis is a complex multiple system disease, which is characterized by different clinical manifestations involving both extraocular and ocular sites. Infectious causes of uveitis accounts for a minority of cases, while idiopathic/non-infectious/autoimmune uveitis, which accounts for the majority, is sometimes associated with systemic diseases (Rosenbaum et al., 2016). Although the exact pathogenesis of uveitis is unclear, accumulating evidence shows that the combination of certain genetic or epigenetic factors causes an imbalance in the regulation of the immune response, leading to the development of uveitis. Epigenetics is a rapidly expanding scientific field, and the research on epigenetic regulation of chronic diseases is rising. This work focused on the epigenetic mechanisms that regulate several autoimmune uveitis, aiming to provide new therapeutic ideas for this field. However, the epigenetic mechanisms of uveitis are still in their early stages. Identifying the spectrum of epigenetic changes in all cells contributing to the pathogenesis of uveitis, especially T cells and neutrophils, and translating the correlation of epigenetic changes into cellular and molecular pathways of uveitis is the main direction forward for the field. Epigenetics and its regulators bring new hope for the treatment of autoimmune uveitis, however, the research in this area is still in the stage of animal model, and its clinical application is still limited; thus, more profound research is warranted.
